# Bayesian parameter estimation for nonlinear modelling of biological pathways

**DOI:** 10.1186/1752-0509-5-S3-S9

**Published:** 2011-12-23

**Authors:** Omid Ghasemi, Merry L Lindsey, Tianyi Yang, Nguyen Nguyen, Yufei Huang, Yu-Fang Jin

**Affiliations:** 1 Department of Electrical and Computer Engineering, University of Texas at San Antonio, San Antonio, TX, USA; 2 Barshop Institute of Longevity and Aging Studies and the Division of Geriatrics, Gerontology and Palliative Medicine, Department of Medicine, University of Texas Health Science Center at San Antonio, San Antonio, TX, USA

## Abstract

**Background:**

The availability of temporal measurements on biological experiments has significantly promoted research areas in systems biology. To gain insight into the interaction and regulation of biological systems, mathematical frameworks such as ordinary differential equations have been widely applied to model biological pathways and interpret the temporal data. Hill equations are the preferred formats to represent the reaction rate in differential equation frameworks, due to their simple structures and their capabilities for easy fitting to saturated experimental measurements. However, Hill equations are highly nonlinearly parameterized functions, and parameters in these functions cannot be measured easily. Additionally, because of its high nonlinearity, adaptive parameter estimation algorithms developed for linear parameterized differential equations cannot be applied. Therefore, parameter estimation in nonlinearly parameterized differential equation models for biological pathways is both challenging and rewarding. In this study, we propose a Bayesian parameter estimation algorithm to estimate parameters in nonlinear mathematical models for biological pathways using time series data.

**Results:**

We used the Runge-Kutta method to transform differential equations to difference equations assuming a known structure of the differential equations. This transformation allowed us to generate predictions dependent on previous states and to apply a Bayesian approach, namely, the Markov chain Monte Carlo (MCMC) method. We applied this approach to the biological pathways involved in the left ventricle (LV) response to myocardial infarction (MI) and verified our algorithm by estimating two parameters in a Hill equation embedded in the nonlinear model. We further evaluated our estimation performance with different parameter settings and signal to noise ratios. Our results demonstrated the effectiveness of the algorithm for both linearly and nonlinearly parameterized dynamic systems.

**Conclusions:**

Our proposed Bayesian algorithm successfully estimated parameters in nonlinear mathematical models for biological pathways. This method can be further extended to high order systems and thus provides a useful tool to analyze biological dynamics and extract information using temporal data.

## Background

In the past decade, there has been a rapid development in systems biology approaches driven by high-throughput data characterizing regulations of genetic networks, interactions among proteins, and reactions in metabolic pathways. These data usually provide a specific scenario of a biological system which may be compared with an alternative system, for instance, expression levels of biomarkers associated with a disease pattern versus healthy controls. Extending the snapshot-type data to condensed data in a time sequence, which is more suitable for profiling the temporal dynamics, provides insights into the functions and underlying regulating mechanisms of the biological system. To gain these insights, mathematical representations of biological systems established with temporal data are highly desirable.

Establishment of proper mathematical representations requires identification of a suitable model with an adequate framework and structure to determine the parameters in the framework. For the structure identification of a model, extensive research has been carried out and mathematical models have been developed to represent the instantaneous rate of a process as an explicit function of all the state variables *x *∈ *R^n ^*that have a direct influence on the process. In these representations, the rate of change in each variable *x_i_*, is determined by the difference between influx and efflux of the variable (equation 1) and each flux *v_i _*is approximated by a product of power-law functions as shown in equation 2:

(1) $$ẋ=v_{in\mathrm{fl}ux}-v_{efflux}$$

(2) $$v_{i}\left(x\right)=\gamma_{i}\overset{n}{\underset{j=1}{\prod }}x_{j}^{\rho_{ij}},$$

where *γ_i _*represents the rate constant and *ρ_ij _*represents the kinetic order of *v_i _*with respect to *x_j _*[1-3]. These approximations have been widely applied to modeling and analysis of biochemical systems for allowing computational simulation of dynamics and parameter estimation for unknown *γ_i _*and *ρ_ij_*. However, the representations have a low range of accuracy when saturation and cooperativity are represented. To address the reaction rate of enzyme-catalyzed reactions with cooperativity and saturation, a preferred mathematical function is the Hill equation which is described as:

(3) $$v_{i}=\frac{Vu_{i}^{H}}{K+u_{i}^{H}}$$

where *V *denotes the maximal rate, *u_i _*denotes the reaction factor, *K *represents the saturation constant, and *H *denotes the Hill coefficient. The Hill coefficient *H *corresponds to the number of binding sites in the molecule that catalyzes the process [4]. Though there is some disagreement regarding how accurate it is to determine the Hill coefficient *H *by the number of binding sites [5], *H *is generally assumed to be a known constant that can be estimated from experimental data. However, it remains problematic to determine the parameters of *V *and *K*.

For linearly parameterized systems, the least squares method generally gives the optimal estimate of parameters. In addition to the least square approach, an adaptive estimation algorithm serves as a powerful tool to estimate the unknown parameters in ordinary differential equations (ODE) [6-9][10]. For nonlinearly parameterized dynamics, Cao and colleagues have studied the conditions for parameter convergence if the nonlinearly parameterized function satisfies the Lipschitz condition [11]. Qu and colleagues have proposed an adaptive control algorithm for a nonlinearly parameterized system with specific input/control in lieu of requiring the Lipschitz condition with respect to parameters [12]. However, a Hill equation in an ODE does not satisfy Lipschitz condition with respect to the parameter *K *and, generally, it is difficult to apply a continuous control determined by estimated parameters and states of biological systems due to the lack of real time measurements. While estimating the parameters of the Hill equation in ODEs provides accurate prediction of the reactions, it is very difficult to incorporate the continuous evaluation of the states that is needed to better understand the regulation of the biological system. Additionally, it is even more challenging when there is sparse experimental data in discrete time sequences, as is often the case.

Bayesian approaches have been widely used for machine learning, adaptive filters, information theory, and pattern recognition [13-16][17][18]. Specifically, Markov chain Monte Carlo (MCMC) methods have demonstrated to be a powerful inference tool for complex systems raised in behavioral science and computational biology [19,20][21]. MCMC gains its popularity due to its easy implementation, ability to generate statistically samples from a target high dimensional distribution, good inference performance, and convenience for statistical analysis of results. Therefore, it is very promising to apply MCMC methods to estimate parameters in nonlinearly parameterized dynamics.

The aim of this study is to estimate the unknown parameters **θ **using a Bayesian approach in nonlinear ODEs representing a biological system as equation (4):

(4) $$\begin{array}{c} \dot{x}=f(\theta ,x(t),u(t),t),x(t_{0})=x_{0}\\ y(t)=g(x(t))+\varepsilon (t) \end{array}$$

In this representation, *x *∈ *R^n ^*denotes the system's state variables, for instance, the concentrations of biochemical factors, and *x*_0 _is the initial state, *f*(·) is a set of nonlinear functions describing the dynamical property of the biological systems, *u*(*t*) ∈ *R^l ^*is the systems input denoting concentrations of stimuli, and ***θ ***∈ *R^p ^*are parameters that characterize dynamic reactions, *y *∈ *R^n ^*represents the observed data subject to a Gaussian white noise *ε*(*t*) ~ *N*(0,*σ*^2^), *g*(·) represents a measurement function and atypical format will be an identical matrix. We assume we have discrete time series of *y*(*t*), *and u*(*t*) and all parameters in **θ **are positive.

We applied our Bayesian algorithm to estimate unknown parameters in the biological pathways involved in the left ventricle (LV) response to myocardial infarction, which involves inflammatory and fibrotic components typical of a wound healing response. Macrophages begin to infiltrate the LV at day 3 post-MI and are stimulated by interleukin-10 (IL-10) to release transforming growth factor β (TGF-β). In turn, TGF-β stimulates fibroblasts to secrete extracellular matrix components that are necessary for an adequate scar to form. Estimates of the parameters were close to their true value with considerably small estimatiom errors, particularly with regards to small noise variances.

## Methods

The mathematical models represented as ODEs generally lead to continuous solutions, while real observed data are typically discrete in the time domain. To bridge the gap between our mathematical model and the real experimental data, and to predict future samples with available observational data, we first transformed the ODE presentation to difference equations.

### Transformation of differential equations to difference equations

With known parameters ***θ***, solutions of equation (4) can be approximated with the fourth-order Runge-Kutta method as follows:

(5) $$\begin{array}{c} x_{i+1}=x_{i}+\frac{h}{6}\left(k_{1}+2k_{2}+2k_{3}+k_{4}\right)\\ \;\;\;k_{1}=f\left(\theta ,x_{i},u_{i},t_{i}\right)\\ k_{2}=f\left(\theta ,x_{i}+\frac{k_{1}}{2},u\left(t_{i}+\frac{h}{2}\right),t_{i}+\frac{h}{2}\right)\\ k_{3}=f\left(\theta ,x_{i}+\frac{k_{2}}{2},u\left(t_{i}+\frac{h}{2}\right),t_{i}+\frac{h}{2}\right)\\ k_{4}=f\left(\theta ,x_{i}+k_{3},u\left(t_{i}+h\right),t_{i}+h\right) \end{array}$$

where *t_i_*, *i *= 0,1,,2,⋯ denotes different sampling time points and *h *denotes a constant interval between *t*_*i *_*and t*_*i*+1_. Thus, the next step of *x*_*i*+1 _is determined by present value *x_i _*and the weighted average of 4 incremental. Without loss of generality, the measurement function *g*(·) is an identical matrix, i.e. *y_i _*= *x_i _*if there is no measurement noise. The predicted output *y^s ^*at *t*_*i*+1 _can be obtained with all available *y_i_*, *u_i_*, and replacement of ***θ ***with estimated parameters $\hat{\theta }$ as:

(6) $$y_{i+1}^{s}=y_{i}+\frac{h}{6}\left(k_{1}+2k_{2}+2k_{3}+k_{4}\right)$$

Estimations of parameter ***θ ***can be obtained by applying a Bayesian approach as follows.

### Estimation of parameters using a Bayesian approach

The goal of estimating ***θ ***using a Bayesian method is to obtain the posterior distribution *p*(***θ***|**y**), which represents our knowledge about the unknown parameters based on the experimental data ***y***, and it can be expressed as:

(7) $$p\left(\theta |y\right)=\frac{p\left(y|\theta \right)p\left(\theta \right)}{\int p\left(y|\theta \right)p\left(\theta \right)d\theta }$$

where *p*(***θ***) is the *prior *distribution representing our knowledge about the parameter ***θ ***prior to observing the experimental data *y*, *p*(***y***|***θ***) is the likelihood function denoting how likely it is to observe the experimental data set given an estimated ***θ***. Based on the posterior distribution, the unknown parameters ***θ ***can be estimated by the minimum mean square error (MMSE) or the maximum *a posteriori *(MAP) criterions, which estimate ***θ ***by the mean or the mode of the posterior distribution *p*(***y***|***θ***), respectively.

However, since the function *f*(*θ*, *x_i_*, *u_i_*, *t_i_*) is highly nonlinear, the close form expression of *p*(***y***|***θ***) cannot be obtained analytically, hence neither the Bayesian estimates. We resort to the numerical solutions using MCMC and specifically, the Metropolis-Hasting (M-H) algorithm. The MH algorithm provides a scheme for generating random samples from the desired posterior distribution, even though its close form is not available. These random samples can be used with ease to approximate the posterior distribution and calculate various estimates of the unknowns.

The MH algorithm is an iterative algorithm and the steps of the proposed algorithm for model (5) at the (*i+1*)*^th ^*iteration can be summarized as the following:

1) Given the parameter sample ***θ***^*i *^obtained in the *i^th ^*iteration;

2) Draw ***θ***^⋆ ^from the proposal distribution *q*(***θ***^⋆^|***θ***^*i*^) as a proposed sample;

3) Calculate the ratio:

(8) $$\alpha =\frac{p\left(y|\theta^{⋆}\right)p\left(\theta^{⋆}\right)}{p\left(y|\theta^{i}\right)p\left(\theta^{i}\right)}\frac{q\left(\theta^{i}|\theta^{⋆}\right)}{q\left(\theta^{⋆}|\theta^{i}\right)}$$

4) Draw a random sample *U*[0,1] and assign the (*i*+*1*)*^th ^*sample as:

(9) $$\theta^{i+1}=\{\begin{array}{cc} \theta^{⋆} & U\leq \lambda \\ \theta^{i} & otherwise \end{array}$$

where *λ *= *min*{1,*α*}.

With the assumption that all parameters are positive, the proposal distribution to generate ***θ***^⋆ ^is chosen as a Gamma distribution expressed as:

(10) $$Gamma\left(\theta ;\eta ,\beta \right)=\frac{1}{\beta^{\eta }\Gamma \left(\eta \right)}\theta^{\eta -1}e^{-\frac{\theta }{\beta }}$$

Accordingly, the proposal distributions *q*(***θ***^⋆^|***θ***^*i*^) and *q*(***θ***^*i*^|***θ***^⋆^) can be written as:

(11) $$q\left(\theta^{⋆}|\theta^{i}\right)\sim Gamma\left(\theta^{⋆};\eta_{1},\beta_{1}\theta^{i}\right)\sim \frac{1}{{\left(\beta_{1}\theta^{i}\right)}^{\eta_{1}}\Gamma \left(\eta_{1}\right)}\theta^{⋆\eta_{1}-1}e^{-\frac{\theta^{⋆}}{\beta_{1}\theta^{i}}}$$

and:

(12) $$q\left(\theta^{i}|\theta^{⋆}\right)\sim Gamma\left(\theta^{i};\eta_{1},\beta_{1}\theta^{⋆}\right)\sim \frac{1}{{\left(\beta_{1}\theta^{⋆}\right)}^{\eta_{1}}\Gamma \left(\eta_{1}\right)}\theta^{i\eta_{1}-1}e^{-\frac{\theta^{i}}{\beta_{1}\theta^{⋆}}}$$

The second fraction in equation (8) becomes:

(13) $$\frac{q\left(\theta^{i}|\theta^{⋆}\right)}{q\left(\theta^{⋆}|\theta^{i}\right)}={\left(\frac{\theta^{i}}{\theta^{⋆}}\right)}^{2\eta_{1}-1}e^{\frac{1}{\beta_{1}}\left(\frac{\theta^{⋆}}{\theta^{i}}-\frac{\theta^{i}}{\theta^{⋆}}\right)}$$

In a real application, there are unavoidable statistical and model noise, which is modeled in this case by the i.i.d. Gaussian distribution with the unknown noise variance *σ*^2^. Therefore, in equation (8), *p*(***y***|***θ***) is the marginal likelihood by integrating the noise variance *σ*^2 ^from the complete likelihood function *p*(***y***|***θ***,*σ*^2^), i.e.,

(14) $$p\left(y|\theta \right)=\int p\left(y|\theta ,\sigma^{2}\right)p\left(\sigma^{2}\right)d\sigma^{2}$$

where *p*(*σ*^2^) is the prior distribution taken to be the conjugate Inverse Gamma (IG) distribution (*IG*(*η*_2_, *β*_2_)) as:

(15) $$p\left(\sigma^{2}\right)\sim IG\left(\eta_{2},\beta_{2}\right)\sim \frac{\beta_{2}^{\eta_{2}}}{\Gamma \left(\eta_{2}\right)}{\left(\sigma^{2}\right)}^{-\eta_{2}-1}e^{\left(-\frac{\beta_{2}}{\sigma^{2}}\right)}$$

and *p*(***y***|***θ***, *σ*) is the product of a series of independent Gaussian distributions. Both of them have the form $p\left(y_{i}|\theta ,\sigma^{2}\right)\sim N\left(y_{i}-y_{i}^{s},\sigma^{2}\right)$, where ***y ***is the experimental data and ***y***^*s *^can be computed using the classical Runge-Kutta method as shown in equation (6). In this relation, given the total number of the observations being *m*, the integration (14) can be expressed as:

(16) $$p\left(y|\theta \right)=\int {\left(2\pi \right)}^{-\frac{m}{2}}{\left(\sigma^{2}\right)}^{-\frac{m}{2}}\overset{m}{\underset{i=1}{\prod }}e^{-\frac{\left(y_{i}^{s}-y_{i}\right)}{2\sigma^{2}}}\frac{\beta_{2}^{\eta_{2}}}{\Gamma \left(\eta_{2}\right)}{\left(\sigma^{2}\right)}^{-\eta_{2}-1}e^{-\frac{\beta_{2}}{\sigma^{2}}}d\sigma^{2}.$$

With the definition of model error as $M.E.=\sum_{i=1}^{m}{\left(y_{i}^{s}-y_{i}\right)}^{2}$, we can have

(17) $$\overset{m}{\underset{i=1}{\prod }}e^{-\frac{\left(y_{i}^{s}-y_{i}\right)}{2\sigma^{2}}}=e^{-\frac{\sum_{i=1}^{m}{\left(y_{i}^{s}-y_{i}\right)}^{2}}{2\sigma^{2}}}=e^{-\frac{M.E.}{2\sigma^{2}}}$$

Now, define new shape and scale factors of the IG function by $\eta_{2}=\frac{\eta_{3}}{2}$ &$\beta_{2}=\frac{\beta_{3}}{2}$ and substitute them in equation (16), we obtain the following equation

(18) $$\begin{array}{llll} P\left(y|\theta \right) & =\int {\left(2\pi \right)}^{-\frac{m}{2}}{\left(\sigma^{2}\right)}^{-\frac{m}{2}}e^{-\frac{M.E^{2}}{2\sigma^{2}}}\frac{{\left(\frac{\beta_{3}}{2}\right)}^{\frac{\eta_{3}}{2}}}{\Gamma \left(\frac{\eta_{3}}{2}\right)}{\left(\sigma^{2}\right)}^{-\frac{\eta_{3}}{2}-1}e^{-\frac{\beta_{2}}{2\sigma^{2}}}d\sigma^{2}\; & & \;\\ & ={\left(2\pi \right)}^{-\frac{m}{2}}\frac{{\left(\frac{\beta_{3}}{2}\right)}^{\frac{\eta_{3}}{2}}}{\Gamma \left(\frac{\eta_{3}}{2}\right)}\int {\left(\sigma^{2}\right)}^{-\frac{m+\eta_{3}}{2}-1}e^{-\frac{\frac{M.E+\beta_{3}}{2}}{\sigma^{2}}}d\sigma^{2}\; & & \;\\ & \; & \end{array}$$

Define $n^{\prime }=\frac{m+\eta_{3}}{2}$, $\beta^{\prime }=\frac{M.E.+g_{3}}{2}$ &*σ*^2 ^= *τ *then the integral in equation can be rewritten as:

(19) $$\int {\left(\sigma^{2}\right)}^{-\frac{m+\eta_{3}}{2}-1}e^{-\frac{\frac{M.E+\beta_{3}}{2}}{\sigma^{2}}}d\sigma^{2}=\int \tau^{-\eta^{\prime }-1}e^{-\frac{\beta^{\prime }}{\tau }}d\tau =\frac{\Gamma \left(\eta^{\prime }\right)}{{\left(\beta^{\prime }\right)}^{\eta^{\prime }}},$$

which is the integral of an IG-type function. Therefore, the expression of the marginal likelihood function can be expressed as:

(20) $$p\left(y|\theta \right)=\frac{{\left(\frac{\beta_{3}}{2}\right)}^{\frac{\eta_{3}}{2}}}{{\left(2\pi \right)}^{\frac{m}{2}}}\frac{\Gamma \left(\frac{m+\eta_{3}}{2}\right)}{\Gamma \left(\frac{\eta_{3}}{2}\right)}{\left(\frac{M.E.+\beta_{3}}{2}\right)}^{-\frac{m+\eta_{3}}{2}}$$

Substituting equations (13) and (20) into equation (8) results in:

$$\alpha =\frac{p\left(y|\theta^{⋆}\right)p\left(\theta^{⋆}\right)}{p\left(y|\theta^{i}\right)p\left(\theta^{i}\right)}\frac{q\left(\theta^{i}|\theta^{⋆}\right)}{q\left(\theta^{⋆}|\theta^{i}\right)}$$

i.e.

(21) $$\alpha ={\left(\frac{M.E.^{⋆}+2\beta_{2}}{M.E.^{i}+2\beta_{2}}\right)}^{-\frac{m+2\eta_{2}}{2}}\frac{p\left(\theta^{⋆}\right)}{p\left(\theta^{i}\right)}{\left(\frac{\theta^{i}}{\theta^{⋆}}\right)}^{2\eta_{1}-1}e^{\frac{1}{\beta_{1}}\left(\frac{\theta^{⋆}}{\theta^{i}}-\frac{\theta^{i}}{\theta^{⋆}}\right)}$$

The above proposed MH algorithm will be run for many iterations until convergence and the samples obtained after convergence are considered samples from the posterior distribution *p*(***θ***|***y***). The span of iterations until convergence is referred to as the burn-in period. Suppose that *N *converged samples are collected after the burn-in. Then the Bayesian MMSE estimate can be calculated as the mean of the *N *samples. The confidence of the estimation can be also evaluated with these samples.

## Results

A first order ODE equation was employed in this study to estimate the unknown parameters in a nonlinear mathematical model. This ODE was originally established to describe temporal profiles of TGF-β post-MI. After MI, the major sources of TGF-β include activated macrophages and fibroblasts. IL-10 stimulates macrophages to secrete TGF-β and its stimulation effect can be approximated as a Hill equation. Since we are initially interested in the macrophage related function at the early stage and will incorporate the effect of fibroblasts at the later stage, we established the mathematical model as follows:

(22) $$ẋ\left(t\right)=-ax\left(t\right)+\frac{Vu{\left(t\right)}^{2}}{K+u{\left(t\right)}^{2}}M_{\phi }\left(t\right)$$

Where *x *denotes the concentration of TGF-β, *M_ϕ _*denotes the macrophage cell density, *u*(*t*) denotes the concentration of IL-10 post-MI, parameter *a *denotes the degradation rate of TGF-β, the maximum activation rate of macrophages by IL-10 and the secretion rate of macrophages for TGF-β are combined together and denoted as the maximum reaction rate as V, and the saturation rate for macrophage activation is denoted as K. The temporal profiles of IL-10 and macrophage infiltration post-MI are determined by the published experimental results in C57 mice [21,21,21,21]. In our computational simulation, parameter *a *= 15 is calculated by the half-life data [21], *x*_0 _= 0.21 is the concentration of TGF-β measured in healthy adult mice before MI. Both V and K are the unknown parameters to be estimated.

A stationary Markov chain was generated by following the proposed MH algorithm. Only samples after the burn-in are retained. Since no prior information about the parameters is available, a flat gamma distribution is chosen for the prior distribution of ***θ***. The values of scale parameter for both linear and nonlinear parameter (V and K respectively) is 2 (*η *= 2); for the shape parameter (*β*), two different values were chosen for linear and nonlinear parameters. This is due to the different range of values that each of them is covering. So for the linear parameter as *V *∈ [0.01, 10], a shape parameter of *β *= 4 is selected. On the other hand, when *K *∈ [1 1000], a proper parameter factor would be *β *= 2000. The proposed distribution for *p*(***θ***^⋆^|***θ***^i^) and *q*(***θ***^*i*;^|***θ***^⋆^) follows the Gamma distribution defined by the same parameters as mentioned earlier (equations 11& 12). The variance of the additive noises follows an Inverse-Gamma distribution defined by parameters *η*_2 _= 2, *and β*_2 _= 10, which are chosen to model the non-informative prior knowledge about the variance. All simulations were run for 2000 iterations and the first 1500 were considered burn-in and removed. The 500 samples after the burn-in of each run were averaged as an MMSE estimate.

**Figure 1:**
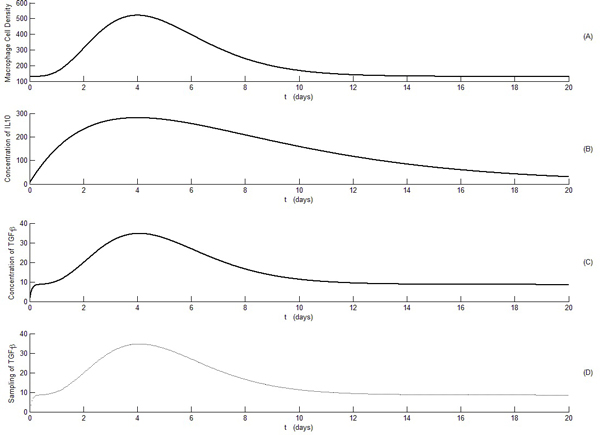
**Temporal profiles of macrophage numbers, IL-10 concentrations, and TGF-β concentrations post myocardial infarction establish the nonlinear dynamic patterns of the MI response**. A: Temporal profile of macrophage infiltration (cell numbers/mm^2^) post myocardial infarction reconstructed from experimental data in C57 mice [21]. B: Temporal profile of IL-10 concentrations (pg/ml) post myocardial infarction reconstructed from experimental data in C57 mice [19,20]. C: Temporal profile of TGF-β computed based on the nonlinear dynamics equation (24). D: Sampled TGF-β profile in part C to represent the sparse experimental data on TGF-β concentrations.

We have simulated three situations: 1) Estimate parameter V with known parameter K; this allows us to evaluate the performance of linear parameterized system using the Bayesian approach. 2) Estimate parameter K given a known V; this allows us to evaluate the performance of estimating a single parameter in nonlinearly parameterized dynamics. 3) Estimate both V and K using the proposed Bayesian approach. To mimic the real experimental data, we sampled our computed state at 500 time points. The temporal profiles of macrophage cell density, IL-10 concentrations, TGF-β concentrations, and sampled TGF-β (500 samples over 20 days) in the time sequence were shown in Figure 1.

### Estimate parameters in linear parameterized system

**Figure 2:**
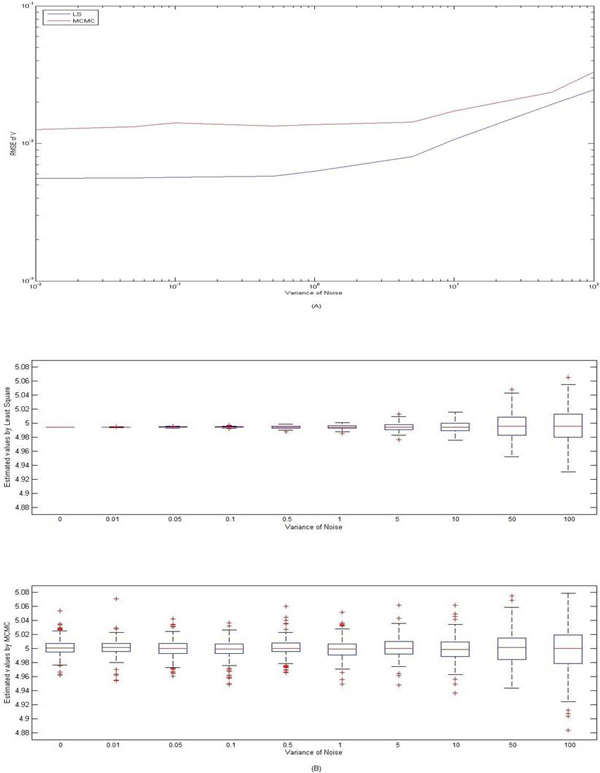
**Performance evaluation for linear parameter estimation**. A: Root mean square error of parameter V given parameter K = 2 was plotted while variance of the noise increased from 0.01 to 100 with both MCMC and least square. The true value of V was set as 5. Root mean square error monotonically increased as variance increased. The root mean square error was calculated as 0.0331 and 0.0256 as variance of noise increased to 100 for MCMC and least square, respectively. This demonstrated that the linear parameter estimation performed within the recommended range. B: the boxplot for the estimation of V were shown for both least square and MCMC methods as the noise variances increased from 0 to 100. The number of outliers is significantly higher for MCMC comparing to least square.

In a Hill-equation, the parameter V is linearly parameterized. In the first set of our simulations, we set K = 2 and estimate the parameter V with the temporal data. The nominal value of V is 5, and the estimated V using MCMC ranges from 4.9247 to 5.0045. The performance of the estimation algorithm can also be evaluated by examining the mean squared error (RMSE) of V with respect to the variance *σ*^2 ^of the noises as shown in Figure 2. RMSE of V increases monotonically as variance *σ*^2 ^increases but remains in a very small region. RMSE of V was calculated as 0.017 while the variance *σ *was increased to 1, suggesting that the estimate of V remains accurate when signal to noise ratio gets lower. This performance demonstrated that MCMC worked very well for linearly parameterized dynamics.

At the same time, the performance of MCMC was compared with least square algorithm. As parameter V to be estimated is a linear parameter, this comparison will give us a good idea about the performance of MCMC. It is expected that the least square gives the best results in estimating the linear parameter with the presence of noise which is shown in Figure 2. There exist large differences between the least square and MCMC algorithms when the variance of noise (*σ*^2^) is small. As the variance of noise increases, the error difference decreases. However it should be mentioned that the outliers in MCMC estimation is significantly more than least square. Same as MCMC estimation, the nominal value of V is 5, and the estimated V using least square ranges from 4.9308 to 5.0561.

### Estimate parameters in nonlinearly parameterized dynamics

To verify our algorithm for nonlinearly parameterized dynamics, we estimated parameter K assuming V available and parameters V and K when both are unknown.

**Figure 3:**
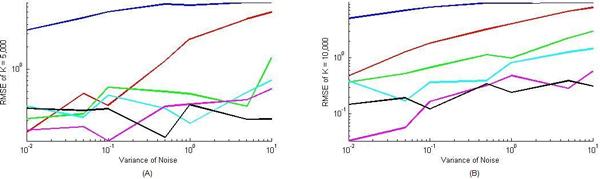
**Performance evaluation for nonlinearly parameterized situation with known V**. Root mean square error of parameter K (A: K = 5000, and B: K = 10000) was plotted with respect to different noise variances ranging from 0.01 to 10 and different values of V. Colors of the curves denote different parameter settings of parameter V. (Blue: V = 0.01, Red: V = 0.1, Green V = 0.5, Cyan: V = 1, Magenta: V = 5, and Black: V = 10).

When a nominal value of K is set as 5000, we ran 6 groups of simulations according to 6 different parameter settings for V (V = 0.01,0.1, 0.5, 1, 5 and 10). Output of each group was subject to white noises with different variances ranging from *σ*^2 ^= 0 *to σ*^2 ^= 10. We repeated such simulation at K = 1, 10, 50, 100, 500, 1000, 5000 & 10000, respectively, and showed our RMSE error of estimated values of V subject to different variances while V = 1 and V = 10 in Table 1. To illustrate the accuracy of the estimation, we also depicted the RMSE of estimated K with different setting of V and variances in Figure 3. Our results demonstrated that the estimated parameter error with the proposed algorithm decreases by increasing the values of V. It was also shown that by increasing the value of K, the parameter can be estimated with less error.

**Table 1:** Estimated values of parameter K subject to different noise variances.

		Estimated value of K				
		
Parameter V	True value of K	σ^2^= 0	σ^2 ^= 0.1	σ^2^= 0.5	σ^2 ^= 1	σ^2 ^= 10
V = 1	K = 1	0.895860924	1.198924271	2.060871616	2.392641064	2.2417669

V = 1	K = 10	9.942624719	9.417099524	5.18899955	3.041348517	2.3984733

V = 1	K = 50	49.84538377	50.3653591	49.79822957	48.21724047	17.612567

V = 1	K = 100	99.57735594	100.5475019	98.86122817	95.19722428	35.651171

V = 1	K = 500	499.7813772	498.9833568	495.3510638	501.8771493	450.50892

V = 1	K = 1000	999.2728579	997.8776983	995.7044492	988.870943	891.65597

V = 10	K = 1	0.970450637	1.006078611	1.094535769	1.282321201	2.1026316

V = 10	K = 10	9.975237184	9.997279446	9.847326837	9.531280999	5.3013845

V = 10	K = 50	50.05571987	49.87267536	49.91859144	50.0966008	49.055078

V = 10	K = 100	99.87386748	99.92057687	100.6109818	99.56079265	97.374093

V = 10	K = 500	500.0809047	499.7714225	499.802065	500.2063382	497.87452

V = 10	K = 1000	999.5597507	999.5670495	1000.211577	1001.028307	996.15873

Parameter V was set to 1 and 10, respectively

**Figure 4:**
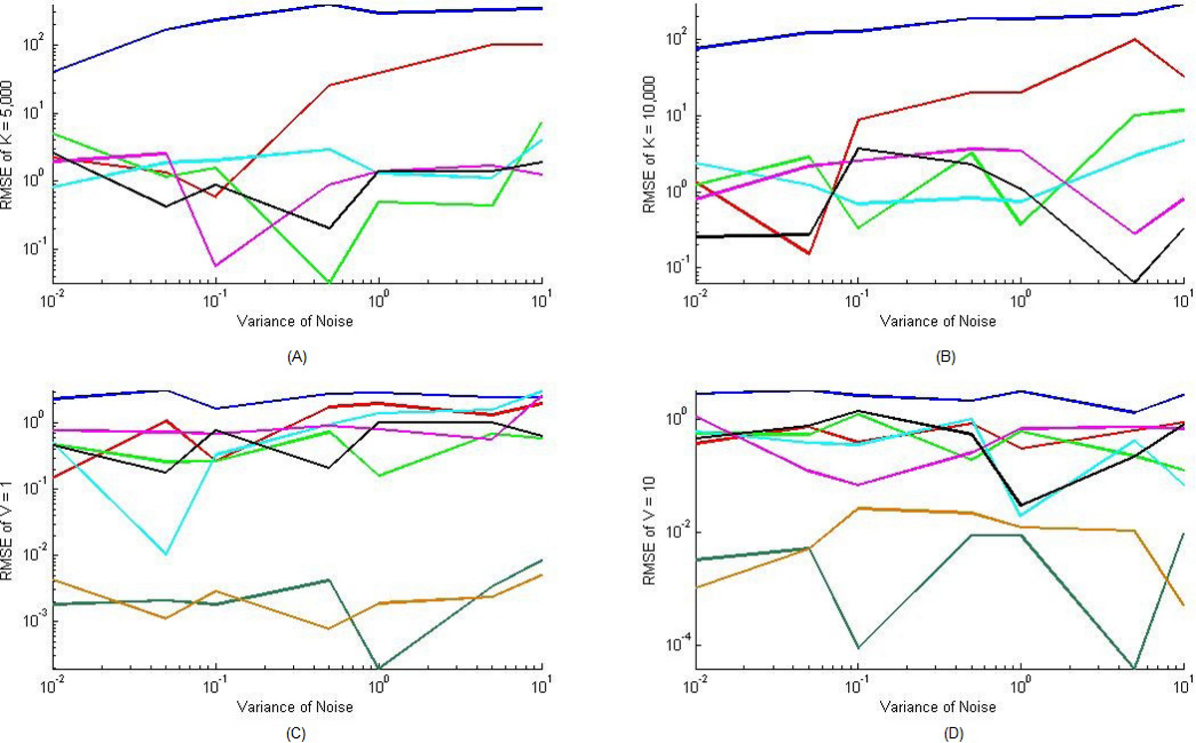
**Performance evaluation for nonlinearly parameterized situation with unknown V and K**. Root mean square error of parameter K was plotted with respect to different noise variances ranging from 0.01 to 1 and different values of V in A and B. (A: true value of K = 5000, and B: True value of K = 10000). In subfigures A and B, colors of the curves denote different parameter settings of parameter V (Blue: V = 0.01, Red: V = 0.1, Green V = 0.5, Cyan: V = 1, Magenta: V = 5, Black: V = 10). Root mean square error of parameter V was plotted with respect to different noise variances ranging from 0.01 to 1 and different values of K in C and D (C: true value of V = 1, and B: True value of V = 10). In subfigures C and D, colors of the curves denote different parameter settings of parameter K (Blue: K = 1, Red: K = 10, Green K = 50, Cyan: K = 100, Magenta K = 500, Black K = 1000, Dark Green K = 5000, Brown K = 10000).

In case that both V and K are unknown, we also ran twenty different settings of the parameters and verified the error of the estimation. We verified the cases while the true value of K was 5000 and 10000, and true value of V was 0.01, 0.1, 0.5, 1, 5, and 10, and while the true value of V was set as 1 and 10, and true value of K was set as 1, 10, 50, 100, 500, 1000, 5000 and 10000. Again, our algorithm generated estimates close to the nominal values and the RMSE of different parameter V when K = 5000 was shown in Figure 4(A), RMSE of different parameter V while K = 10000 were shown in Figure 4(B), RMSE of different parameter K while V = 1 was shown in Figure 4(C), and RMSE of different parameter K while V = 10 was shown in Figure 4(D), respectively. It can be seen from our results in Figure (4A and 4B) that the error of estimated parameter decreases when the values of V increased. It was also demonstrated that when K increases, a better estimation of this parameter can be achieved. It can be seen in Figure (4C and 4D) that when parameter K increases, K can be estimated with less error for any particular value of linear parameter V. There is not a huge difference between the RMSE of the estimated parameter when increasing V from 1 to 10. In general, RMSE of parameters increased as variances of the noises increased, and RMSE of parameter K was greater than RMSE of parameter V.

## Discussion

This study is the first investigation to estimate unknown parameters in nonlinearly parameterized biological dynamics using MCMC algorithm. We have applied a Bayesian approach to estimate two unknown parameters in an ODE model describing the temporal profiles of TGF-β in the post-MI setting. Our computational results have demonstrated the effectiveness of the Bayesian approach for parameter estimation in a nonlinear model for biological pathways. As such, this study provides a valid estimation approach for nonlinear dynamics of biological pathways. The most important contributions of this study are listed as follows: 1) The new proposed method bridges the gap between the sparse observational data and the need for continuous signals embedded in mathematical models. Therefore, parameters estimated on the basis of experimental data have clear biological meaning in the mathematical models. 2) The introduction of additive noises and measurement functions reflect real scenarios in biological experiments, therefore, giving more confidence to the parameter estimation real world in applications. 3) A new MCMC algorithm is proposed to estimate parameters in general nonlinearly-parameterized dynamics. Our results demonstrated good performance in estimating two parameters of an ODE with a Hill equation. Together, this new method will have widespread applicability to many biological systems, not limited to investigations on cardiovascular disease.

In this study, our key task is defined as parameter estimation for a nonlinearly parameterized mathematical model for biological pathways. As there exist different representations of mathematical models such as linearized models and power law functions, it is possible to approximate nonlinearly parameterized dynamics by linearly parameterized dynamics [21], which would significantly reduce the difficulty for parameter estimation. However, it is worth noting that transformations to a linearized model and power law modelonly guarantee 1) the transformed models are identical to the original Hill representation at the operating point *u*_0_; and 2) the transformed model have the identical first-order derivatives at the operating point *u*_0 _as the original Hill representations. Therefore, both linear and power low approximations hold locally in a small vicinity of the operating point *u*_0_. When the variable, *u*, in Hill representation has large variations, in reality, these transformations may lead to huge modeling errors. Though these transformations will greatly reduce the complexity of parameter estimation, they cannot provide accurate estimation. This emphasizes the necessity of parameter estimation in nonlinearly parameterized dynamics directly and our proposed MCMC algorithm is a response to this need.

While we illustrated the effectiveness of the algorithm with a first order ODE model, the algorithm can be expanded to estimate more parameters with higher order ODE models for more complicated systems. In that case, convergence of the estimates and convergence speed of the algorithm should be further studied. Additionally, the measurement function we used in this study is an identical matrix, this identical matrix can be relaxed by an observable function where all states *x *can be reconstructed by the output *y*.

In this study, we proposed flat Gamma distributions as the proposal distributions in the MH algorithm. Although they lead to implementations with relatively slow convergence of Markov chains, the algorithm can still produce very robust estimation results. Selection of better proposal distributions that will lead to faster convergence, thus more efficient implementation of the algorithm will be a focus of our future study. In addition, we employed real experimental data in this study to estimate the effects of IL-10 on TGF-β concentrations in left ventricle post-MI and our measurement equation includes additive noises to simulate the real biological systems. However, we are well aware that the structure of the model is simplified and there exist modeling errors embedded in the structure of the mathematical model. These modeling errors will likely lead to estimation errors of the parameters. We can minimize the modeling error with the accumulation of more biological knowledge. Though it is beyond the scope of the current paper, further investigation on modeling structure using real *in vivo *experimental results has been planned for our future research.

## Conclusions

In conclusion, we have proposed an algorithm which combines the transformation from ODEs to difference expressions and a Bayesian algorithm to estimate multiple parameters in a nonlinear mathematical model for biological systems using discrete observational experimental data. Estimates of the parameters were close to their true values with considerably small estimation errors, particularly with regard to small noise variances. This proposed estimation algorithm provides a powerful tool to analyze time series data and better understand the interactions among biological pathways.

## Authors' contributions

YFJ, and YH designed the research; OG, TY and NN performed computational simulation. OG, TY, MLL, YH., and YFJ analyzed the results and wrote the manuscript. All authors have read and approved the final manuscript.

## Competing interests

The authors declare that they have no competing interests.
